# The associations of decent work with wellbeing and career capabilities: a meta-analysis

**DOI:** 10.3389/fpsyg.2023.1068599

**Published:** 2023-04-13

**Authors:** Xuebing Su, Ko Ling Chan

**Affiliations:** Department of Applied Social Sciences, The Hong Kong Polytechnic University, Kowloon, Hong Kong SAR, China

**Keywords:** decent work, wellbeing, career capabilities, career development, meta-analysis, psychology of working theory, global sustainable development, agency-structure theory

## Abstract

**Introduction:**

As a global sustainable development goal, the decent work notion has been promoted all over the world at theoretical, practical, and research levels for the purpose of enhancing people's capacity to enjoy freedom, equity, security, and human dignity at work. However, conclusive findings of the impact of decent work on people's wellbeing and longer-term career development are still missing due to a lack of systematic reviews on this topic. This study aims to (a) investigate the associations of decent work with people's wellbeing and their capabilities for sustaining career development and (b) examine the differential associations across different subgroups.

**Methods:**

Databases of literature archived on or before 4 March 2022 were searched. A total of 46 studies met the inclusion criteria and were included in the analysis for the combined Pearson correlation coefficient (r) to estimate the associations of decent work with wellbeing and career capabilities, among which 30 studies (16,026 participants) were used for calculating the association between decent work and wellbeing whereas 26 studies (12,384 participants) were used for decent work and career capabilities.

**Results and discussion:**

First, decent work demonstrates a medium association with wellbeing (*r* = .48, 95% CI [.45, .51]), and a medium association with career capabilities (*r* = .44, 95% CI [.40, .49]). Second, no significant differences with respect to the association of decent work with wellbeing and career capabilities were identified across subgroups categorized by developed/developing countries, population type, social status of participants as employee or student, participants from vulnerable/general groups, aspects of wellbeing/career capabilities, and study design. These results pose important implications for informing future research and practice to measure and promote decent work across the world.

## Introduction

Decent work as a global agenda for promoting social justice in social, political, and economic development has attracted increasing academic interest in the past 20 years (International Labor Organization., 2002; Bellace, 2011; Brill, 2021; Silva, 2021). In 1999, the International Labor Organization (ILO) coined the term decent work as the sum of people's work-related aspirations, and later started promoting decent work all over the world to guide the assessments of working conditions at both structural and individual levels that can be counted as decent including, but not limited to, union density, occupational safety, legal protection for workers, availability of social security, etc. (International Labor Organization, 2012). Since its emergence, there has been substantial academic discussion on the uniqueness of the decent work construct by comparing it with relevant concepts, including unemployment, work-life balance, career management, etc., and a systematic review concluded that these concepts have contributed to a specific dimension of decent work, and their functions are confined to informing certain actions for pursuing sustainable development (Pereira, 2019). A recent systematic review (Stefana et al., 2021) on the measures of quality of working life restricted to European contexts has highlighted using composite indicators to describe work conditions. This is considered trendy as it reduces the complexity of using many sparse dimensions, and enables more efficient comparisons across different professions and cultural backgrounds and can thus inform better decision-making at the policy level (Stefana et al., 2021). Nevertheless, the concept of quality of working life and its synonyms for describing both objective and subjective work conditions in a composite manner still cannot replace the concept of decent work.

The notion of decent work has been granted legitimacy as a distinctive concept for three reasons: First, decent work is proposed by the ILO as a new lens for reviewing work conditions, which is enlisted as one of the important goals to call for collective actions to promote global sustainable development (Bellace, 2011; Silva, 2021). Second, the decent work notion is developed with the Decent Work Agenda (DWA), which consists of 11 substantive elements (International Labour Organization., 2008; International Labor Organization, 2013) and aims to realize four main values underlying the ILO's actions, namely freedom, equity, security, and human dignity (International Labor Organization., 1999; p. 3). Decent work is perceived as the lever for expressing these four values by means of pursuing four strategic objectives: (1) standards and fundamental principles and rights at work, (2) employment, (3) social protection, and (4) social dialogue (International Labor Organization., 1999; International Labour Organization., 2008). Nowadays decent work has been promoted all over the world at theoretical, practical, and research levels (Pereira, 2019; Rantanen et al., 2020; Blustein et al., 2023) and applied to various population groups from high-skilled occupations (e.g., knowledge workers like college/university faculty members) to low-skilled ones (e.g., domestic workers) (D'Souza, 2010; Ferraro et al., 2021). Third, as a lens for informing a comprehensive review of working conditions, the decent work notion is conceptualized as a multidimensional concept encompassing various favorable or enabling working dimensions. Fourth, the decent work concept differs from other concepts such as quality of working life, as it is also characterized by a future perspective (Brill, 2021). That is also the reason why decent work is suggested to be regarded as aspired dimensions of work rather than as immediately achievable goals (Webster et al., 2015). How people make sense of their work-related aspirations in their specific contexts is perceived to influence their present and future wellbeing (Su et al., 2022). Overall speaking, decent work can be understood as an ideal state of current work conditions as well as a work-related aspiration at the personal level, and a policy objective at the macro level.

Empirical studies on decent work in organizational contexts have increased dramatically in the past few years since the emergence of some psychometrically sound instruments. Webster et al. (2015) developed a diagnostic tool with nine indicators identified by the ILO for studying relatively underprivileged workers such as security guards, farm workers, and hospitality workers in South Africa. Using a sample of knowledge workers from Portugal and Brazil, Ferraro et al. (2018) developed the Decent Work Questionnaire (DWQ), which consists of 31 items and seven dimensions, namely fundamental principles and values at work, adequate working time and workload, fulfilling and productive work, meaningful remuneration for the exercise of citizenship, social protection, opportunities, and health and safety. Duffy et al. (2017) developed the Decent Work Scale (DWS) among working adults in the United States, which consists of 15 items and five dimensions, namely safe working conditions, access to healthcare, adequate compensation, free time and rest, and complementary values. Duffy's DWS has also been widely validated across different cultural backgrounds, including Italy, Switzerland, the UK, Brazil, and Korea, etc. (Di Fabio and Kenny, 2019; Dodd et al., 2019; Masdonati et al., 2019; Nam and Kim, 2019; Ribeiro et al., 2019). Riding on the conceptualization of decent work as a concept to be integrated into the assessment of current work conditions and as a work-related aspiration, and the existing operationalizations of the concept, this study aims to search for meta-analytic evidence regarding the associations of decent work with two other important concepts in relation to people's career and life development, namely wellbeing and career capabilities. The emphasis of the concept of wellbeing is placed more on understanding the current state of people's life; whereas the concept of career capabilities emphasizes the psychosocial resources and constraints affecting people's sustainable career development. Conclusions derived from a first-ever meta-analysis regarding the associations of decent work with wellbeing and career capabilities are expected to justify the promotion of decent work as a global sustainable development goal for the sake of improving people's current state of life as well as sustaining their long-term development.

## Decent work and people's wellbeing

The association of decent work with people's wellbeing or current state of wellbeing is an important research topic which is related to examining the extent to which decent work influences people's general wellbeing. As an overarching concept, wellbeing can be classified into three different aspects, the first of which is positive wellbeing such as life satisfaction, work satisfaction, happiness, etc.; the second is negative wellbeing such as burnout, fatigue, stress, etc., and the final one is needs satisfaction which denotes a wide spectrum of human needs, such as survival needs, social connection needs, and self-determination needs, etc. Identified as a set of favorable working conditions, decent work has been conceptually assumed to positively influence people's holistic wellbeing and this hypothesis has been supported by empirical studies conducted in different countries and among different population groups. For example, Allan et al. (2020) have revealed that decent work was moderately associated with meaningful work (effect size = 0.54) among a sample of 1,069 workers sampled from a wide spectrum of occupational backgrounds in the United States. Atitsogbe et al. (2021) have revealed that decent work exhibited a mild association with job satisfaction and life satisfaction (effect sizes are.33 and.29 respectively) among 334 teachers from Africa. Di Fabio and Kenny (2019) have found that decent work is moderately associated with occupational fatigue and turnover intention (respective effect size = 0.55 and.44). McIlveen et al. (2021) have revealed a relatively lower association between decent work and turnover intention with an effect size of.33 among 201 general workers from Australia. Duffy et al. (2019) have reported that decent work relates strongly to self-determination needs satisfaction (effect size = 0.76), whereas Huang et al. (2021) have reported a relatively low level of association of decent work and needs satisfaction among 434 general workers in China.

However, this sparse evidence base is not able to provide a precise estimate of the correlation between decent work and people's wellbeing. It is also unknown whether the generalizability of the results of these individual studies are biased by any other factors such as the variety of population types, and socioeconomic and political backgrounds. These research gaps are deemed unfavorable to committing global resources and actions to achieving decent work.

## Decent work and career capabilities for sustainable development

The association of decent work with career capabilities is another important concern which is usually studied in parallel with the topic about the association of decent work with wellbeing, particularly as pursuing decent work as a goal of sustainable development is not just for the purpose of improving the current life of people but also for enhancing their longer-term career development. The term “career capabilities” is proposed in accordance to the capability approach developed by Amartya Sen as a humane approach to promoting economic and welfare development (Sen, 1993, 2001, 2008). The concept of capabilities refers to what people will be able to do given that opportunities and resources are available, which is distinguished from the concept of “functionings” understood as achieved abilities (Krishnakumar, 2007). The capability approach challenges the existing normative framework overemphasizing the importance of educational and career credentials and creates space to develop enabling contexts by means of policy making, service delivery and professional practice for promoting people's sustainable development (Orton, 2011; Avis, 2014; Fuertes et al., 2021; Joncas and Pilote, 2021). The capability approach also takes a critical examination of the measures for enhancing people's psychosocial resources and reducing their psychosocial constraints for the purpose of sustaining their own career development (Robertson, 2015; Pouyaud, 2016; Su and Wong, 2022). Career capabilities denote both psychosocial resources and constraints that are subject to one's contexts and are associated with people's psychological, social, and economic status and are expected to influence their sustainable career development. The notion of psychosocial resources covers a wide range of psychosocial characteristics of people that are enabling for people's sustainable career development such as career adaptability (Savickas and Porfeli, 2012), work volition (Duffy et al., 2012), proactivity (Bateman and Crant, 1993), motivation (Dewett, 2007), psychological capital (Luthans et al., 2007), social status as economic resources (Adler et al., 2000), and social support (Zimet et al., 1988), etc. The notion of psychosocial constraints covers a wide range of psychosocial characteristics that may jeopardize people's career development such as economic constraints (Duffy et al., 2016), encounters of marginalization (Duffy et al., 2019), and unemployment history (Rossier and Ouedraogo, 2021), etc.

Highlighting the psychosocial perspective and the sustainability notion, the concept of career capabilities differs from other career-related constructs such as perceived employability, psychosocial employability, sustainable employability, and career competence. The first three terms, respectively, express the notions of abilities, attributes, and conditions enjoyed by a person that allow him or her to be employed, yet each of these three concepts also has its unique features associated with a distinctive qualifier. Specifically, perceived employability highlights one's ability to solve specific work-related problems and handle difficult situations, which is deemed more relevant to one's self-efficacy at work (Berntson et al., 2008); psychosocial employability denotes the psychosocial character of a person's attributes and skills that can enhance their suitability for being employed and for managing contextual changes (Coetzee et al., 2016); and sustainable employability denotes the favorable opportunities and conditions that allow a person to make a valuable contribution through their work in a sustainable manner (Van der Klink et al., 2016). Compared with these three concepts, the concept of career capabilities is less confined to the attributes that are associated with one's achievement of employment or paid work but emphasizes the psychosocial conditions that influence their coping with contextual changes and crisis, and functions in a person's holistic career and life journey, including their engagement in paid work, unpaid work, and non-work domains. The concept of career competence is developed based on the competence approach, which highlights the achieved abilities and functioning of a person that are favorable to their career development, such as self-knowledge, occupational knowledge, planning and decision knowledge, etc. (Dreyfus and Dreyfus, 2005; Ohlemann and Driesel-Lange, 2019). Compared with career competence, the concept of career capabilities highlights the importance of creating an enabling environment by modifying the psychosocial resources and/or psychosocial constraints to support individuals with their lifelong career development. In this connection, informed by the capability approach, the research topic regarding the association of decent work with career capabilities offers the space to discuss the impact of decent work on people's longer-term development as well as to address the changes needed to create an enabling environment for enhancing people's psychosocial resources and reducing their psychosocial constraints (Su and Wong, 2022; Su et al., 2022). Empirical studies have also investigated the association of decent work with different forms of career capabilities in a sparse manner. For example, career adaptability as a manifestation of career capabilities denoting individuals' psychosocial resources for coping with current and anticipated tasks, transitions, and traumas as fostered by the changing conditions with growing awareness and information seeking, and decision-making capability, has been widely studied in the context of promoting decent work. However, the magnitude of the association between decent work and career adaptability varies across different studies. Kim et al. (2019) reported a strong association of decent work with career adaptability among 407 general workers in the United States (effect size = 0.61); Wei et al. (2022) reported a moderate association in a sample of 254 university students from impoverished families in China (effect size = 0.49); England et al. (2020) reported a weak association in a sample of 528 general workers in the United States (effect size = 0.22). Such inconsistent findings were also identified with respect to the association of decent work with other specific career capabilities, leading to an inconclusive estimate of the correlation between decent work and career capabilities.

## A meta-analysis is needed to estimate the associations of decent work with wellbeing and career capabilities

Conclusive findings regarding the associations of decent work with wellbeing and career capabilities and the heterogeneity of the associations across different contexts are in urgent need not only for the promotion of Decent Work Agenda but also for constructing a theory of change to explicate how human beings' holistic wellbeing and career development can be improved by their access to decent work. Against the backdrop that a novel conceptual framework, namely the psychology of working theory (Blustein et al., 2016; Duffy et al., 2016) has prioritized the role played by decent work in facilitating people to pursue wellbeing and self-fulfillment and to counteract the negative effects of individual and social constraints, a precise estimate of the association of decent work with wellbeing and career development is needed to foster the development and verification of this theory.

Despite the research on decent work has been lasting for two decades, empirical studies on decent work are still developing at an early stage. As a systematic review of the impact of decent work in organizational contexts, Pereira (2019) reviewed 38 empirical research studies on decent work published between 2003 and 2017; yet this study did not directly address the association between decent work and career capabilities. So far there is no meta-analysis published on the association of decent work with people's wellbeing or capabilities for sustaining career development. Therefore, this study aims to (a) investigate the associations of decent work with wellbeing and career capabilities, and (b) examine the differential associations across different types of categorization encompassing developed/developing countries, population types, social status of participants as employee or student, participants from vulnerable/general groups, aspects of wellbeing/career capabilities, and study design.

## Method

### Literature search strategy

This study covers publications and unpublished theses in English archived in the electronic databases of EBSCOhost (all databases in EBSCOhost, including EBSCO eClassics Collection, ERIC, MEDLINE, business sources complete, etc.), Scopus, Web of Science, PsycINFO, Embase, PubMed, Emerald, and Scholar Google on or before 4 March 2022. Relevant publications were systematically searched for in all databases except Scholar Google through titles, keywords, and abstracts with “decent work.” Scholar Google was searched through titles with “decent work.”

### Inclusion and exclusion criteria

The eligibility criteria were chosen to be broad in order to include all potentially relevant studies. Studies were included if they fulfilled the following criteria: (1) the study was reported in English, (2) the study quantitatively measured decent work as a distinctive concept, and (3) the study quantitatively reported the results regarding the associations of decent work with at least one indicator of wellbeing or career capabilities of any population groups.

**Figure 1:**
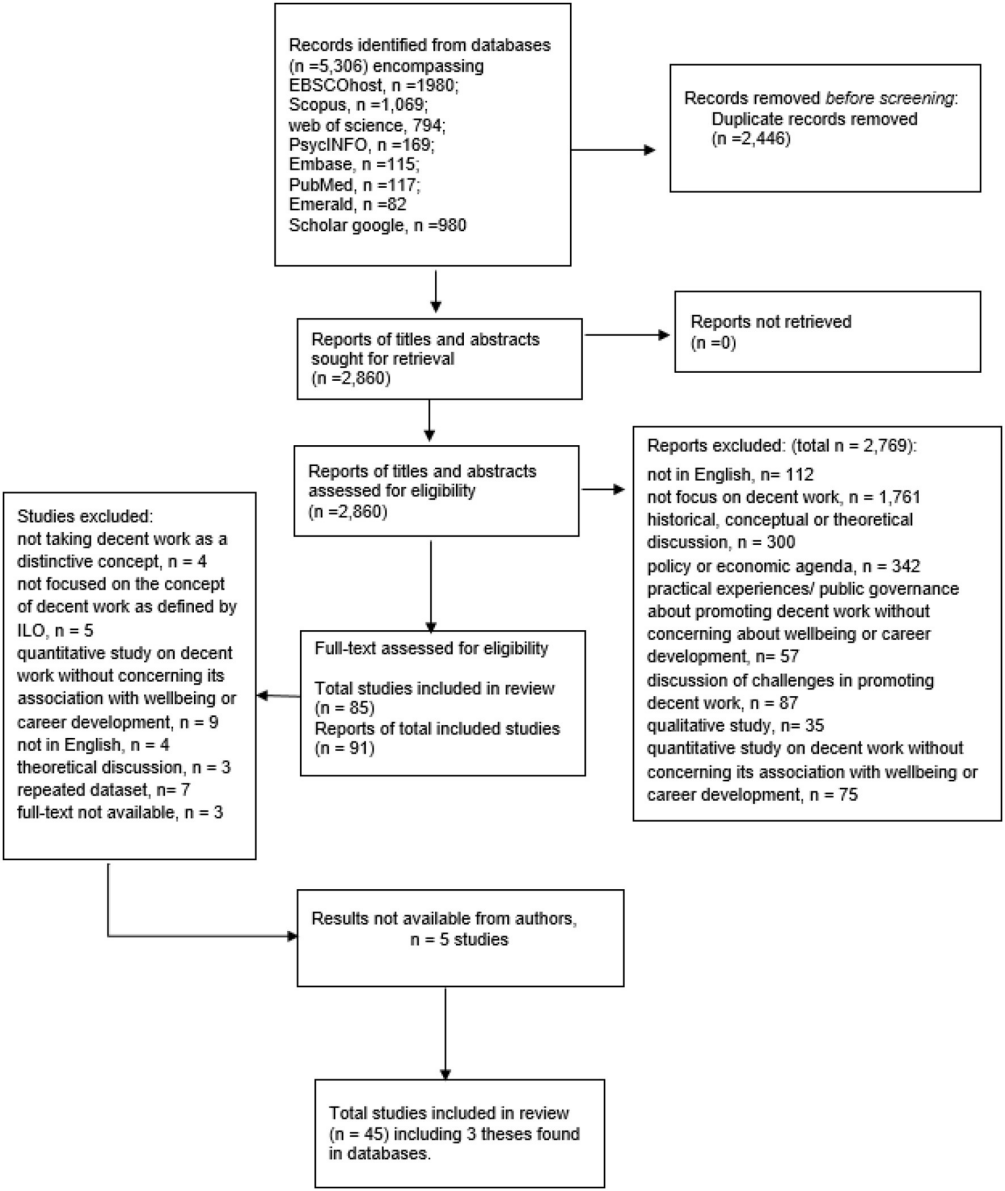
Flow diagram of the study selection.

EndNote bibliographic management software was used to organize the studies. Figure 1 displays the flow diagram of the selection process. The electronic database search yielded 2,860 reports after removing 2,446 duplicate reports with the assistance of electronic sorting using EndNote. Among the 2,860 reports, 2,769 reports were excluded when we reviewed the titles and abstracts, and 35 reports were excluded when we reviewed the full texts, including three reports without accessible full texts. Studies were excluded during title/abstract/full-text reviews if the study was (1) not reported in English; (2) not focused on decent work or did not regard decent work as a distinctive concept; (3) merely focused on a historical, conceptual or theoretical discussion of decent work; (4) merely focused on discussing decent work as a policy or economic agenda; (5) merely focused on the practical experiences or public governance about promoting decent work; (6) merely focused on discussing the challenges encountered in promoting decent work; (7) reported the potential associates of decent work in a qualitative manner, (8) focused on using repeated databases, or (9) did not study associations with any indicators of wellbeing or career capabilities. Another five studies were excluded as the results were not available from the authors.

### Data extraction

The selected studies were reviewed for systematic coding based on a standardized coding form. The following information was extracted from each study: publication information (i.e. author(s), year of publication, and country), methodological characteristics (i.e., study design, instruments for measuring decent work, outcome variables and measures of wellbeing/career capabilities, and use of a psychological perspective), and characteristics of participants (i.e., population groups, gender and age range). For each outcome, the effect size data (i.e., Pearson correlation coefficient (r), which is commonly used by the included studies as a statistical measure for the associations, and sample size) were extracted. To ensure reliability, each study was coded by the first author and a trained research associate independently. The raters typically achieved high interrater agreement of over 95% on coding and those discrepant codes were discussed until the issues were resolved. When a study included more than one independent sample, we classified each sample as a single study and coded the correlation coefficients within the sample separately. When a study reported multiple indicators of wellbeing/career capabilities, we coded all indicators and then averaged them into a single mean value. Notably, as decent work is conceptualized as an overarching concept with multi-dimensions, data which revealed the correlations of any specific dimension(s) in decent work with wellbeing and/or career capabilities were not extracted in this study.

### Statistical analysis

The second version of the software of Comprehensive Meta-Analysis (CMA) was used to conduct statistical analyses. Random-effects models were used in the analyses. To investigate the association of decent work with wellbeing, we first examined the association of decent work with wellbeing as an overarching concept. Then we examined the association of decent work with each of the aforementioned wellbeing variables. Finally, we tested the differential associations across subgroups which were categorized by developed/developing countries, population type, social status of participants as employee or student, participants of vulnerable/general groups, aspects of wellbeing/career capabilities, and study design. Similar data analyses were applied to test the association of decent work with career capabilities. Statistically speaking, all the associations were assessed by the mean correlation coefficients (i.e., Pearson's r). *Q* statistics (i.e., the deviation of each study's effect size from the mean effect) and *I*^2^ (i.e., the percentage of the variability in effect estimates that is due to heterogeneity rather than sampling error) were performed to assess the heterogeneity of effect sizes. Heterogeneity shows a test of the null hypothesis that the true effect size is identical in all included studies and that 100% of the variation in the observed effects is due to sampling error. Moreover, we calculated the prediction interval to predict the true correlation coefficients for 95% of all comparable populations by using the mean effect size, upper limit of confidence interval, Tau^2^ (i.e. the variance of the true effect sizes underlying our data), and number of included studies. Finally, to assess the risk of publication bias, we used a funnel plot to conduct a visual inspection of the data.

## Results

### Characteristics of studies

In total, 46 studies (23,369 participants) met the inclusion criteria and were included in the analysis for the combined Pearson correlation coefficient (r) to estimate the association of decent work with wellbeing and career capabilities, among which 30 studies (16,026 participants) were used for calculating the association between decent work and wellbeing; whereas 26 studies (12,384 participants) were used for decent work and career capabilities. Table 1 presents the study characteristics. The included studies were focused on a wide spectrum of population groups, including workers from diverse occupational backgrounds varying from high-skilled to low-skilled workers, as well as university students. Some included studies were focused on population groups with specific vulnerabilities, including sexual minorities, workers with disabilities, first-generation college students, university students from impoverished families, low-income workers, domestic workers, etc.

**Table 1:** Study characteristics and effect sizes.

**Study name**	**Country^a^**	**Developed/ developing countries^b^**	***N***	**Population types^c^**	**Vulnerable group^d^**	**Students/employees^e^**	**Age range (mean of age; SD)**	**Study design^f^**	**Measure of DW**	**Outcomesindicators^g^**	**Measures foroutcomes**	**Correlation coefficient(s)**
Allan et al. (2019)	U.S.	DEC	364	SM	V	E	18–66	CS	DWS	w10a/c2a	WAMI/WVS	0.58/0.64
Allan et al. (2020)	U.S.	DEC	1069	GW	NV	E	19–74	LS	DWS	w6a/w6b/w10a/w6c	LAMB/WAMI	0.43/0.35/0.54/0.45
Atitsogbe et al. (2021)	Africa	DIC	334	T	NV	E	23–61 (39.30; 7.73)	CS	DWS	w3b/w8a	JSS/SWLS	0.33/0.29
Autin et al. (2019)	U.S.	DEC	476	GW	NV	E	(36.22; 10.76)	CS	DWS	w3b/w8a/w5b	JSS/SWLS/MNSS	0.60/0.40/0.50
Autin et al. (2021)	U.S.	DEC	287	GW	NV	E	19–63 (31.84; 8.38)	CS	DWS	c1a/c8a/c8b/c2a	CAAS/ECS/LEMS/WVS	0.29/−0.32/−0.27/0.50
Bettonville (2019)	U.S.	DEC	285	GW	NV	E	18- 65 (33.36; 9.98)	CS	DWS	c1a/c8a/c8b/c2a	CAAS/FIPT/PM/WVS	0.32/0.20/−0.15/0.45
Bolito (2020)	P and B	Both	738	GW	NV	E	21–80	CS	DWQ	c6a	PCQ	0.44
Buyukgoze-Kavas and Autin (2019)	Turkey	DEC	453	GW	NV	E	19–62 (34.18; 8.45)	CS	DWS	w3b/w10a/w4a	JSS/WAMI/WIS	0.53/0.28/−0.51
Di Fabio and Kenny (2019)	Italy	DEC	436	GW	NV	E	22–65 (43.57; 10.77)	CS	DWS	w3b/w10a/w2d/w4a	OFER	0.51/0.46/−0.55/−0.44
Di Fabio et al. (2021)	Italy	DEC	234	GW	NV	E	(45.05; 11.75)	CS	DWS	w2f	JSS/WAMI/OFER/WIS	−0.46
Dinis (2020)	P and B	Both	727	T	NV	E	21–80 (56)	CS	DWQ	w2a	CBI	−0.48
Douglass et al. (2017)	U.S.	DEC	218	SM	V	E	8–85 (26.41; 7.21)	CS	DWS	c1a/c8a/c8b/c2a	CAAS/SC/HHRDS/WVS	0.29/−0.39/−0.44/0.76
Douglass et al. (2020)	U.S.	DEC	238	GW	NV	E	(32.17; 8.70)	CS	DWS	c1a/c8a/c8b/c2a	CFI/ECS/LEMS/WVS	0.56/0.58/−0.41/0.69
Duffy et al. (2017)	U.S.	DEC	589	GW	NV	E	(35.29; 11.72)	CS	DWS	w3b/w10a/w2d/w4a	JSS/WAMI/OFER/WIS	0.58/0.48/−0.62/−0.51
Duffy et al. (2018)	U.S.	DEC	526	GW	NV	E	(33.58; 8.50)	CS	DWS	c1a/c9a/c8b/c2a	CAAS/HI-SC-SSS/GEDS/WVS	0.24/.31/−0.27/0.67
Duffy et al. (2019)	U.S.	DEC	497	LIW	V	E	(40.40; 13.57)	CS	DWS	w7a/w6d/w5f	WNSS	0.55/0.49/0.53
Duffy et al. (2020)	U.S.	DEC	1540	GW	NV	E	(44.31; 14.36)	LS	DWS	c8a/c8b/c1a/c2a	ECS/LEMS/CFI/WVS	−0.37/−0.37/.34/.58
Duffy et al. (2021)	U.S.	DEC	569	GW	NV	E	NA	LS	DWS	w9a/w9b/w9c/w5f/w2f	FPAQ/PHQ/WNSS/WFI	0.33/−0.57/0.52/0.60/−0.53
England et al. (2020)	U.S.	DEC	528	GWW	NV	E	21–77 (47.21; 12.81)	CS	DWS	c1a/c8a/c8b/c2a	CFI/ECS/LEMS/WVS	0.22/−0.36/−0.37/0.42
Ferraro et al. (2018)	Brazil	DIC	1039	KW	NV	E	21-	CS	DWQ	w2e/w1f	CBI/UWES	−0.52/0.46
Ferraro et al. (2018)	Portugal	DEC	636	KW	NV	E	21-	CS	DWQ	w2e/w1f	CBI/UWES	−0.41/0.33
Ferraro et al. (2021)	Italy	DEC	1465	KW	NV	E	36–50	CS	DWQ	w2e/w1f	CBI/UWES	−0.52/0.51
Ferreira et al. (2019)	Portugal	DEC	345	GW	NV	E	22–74 (44.7; 10.82)	CS	DWS	w1a/w2b/w1c/w2c/w3b/w8a/ w10a/w4a/w1e	UWES/MBI-GS/JSS/SWLS/WAMI/WIS	0.26/−0.40/0.43/−0.52/0.54 /0.45/0.40/−0.40/0.49
Huang et al. (2021)	China	DIC	434	GW	NV	E	NA	LS	DWS	w5a	BNS	0.36
Huang et al. (2022)	China	DIC	568	GW	NV	E	15–28 (18.32;)	CS	DWS	w5c	PSS	0.39
Işik et al. (2019)	Turkey	DEC	326	GW	NV	E	18–60 (30.4; 9.3)	CS	DWS	w3b/w8a/w10a/w4a	JSI/SWLS/WAMI/WIS	0.52/0.52/0.53/−0.43
Kashyap and Arora (2022)	India	DIC	280	FM	NV	E	(33; 6.05)	CS	DWS	w10a/w1f/w6e	WAMI/UWES/WFE	0.40/0.39/0.50
Kim et al. (2019)	Korea	DEC	407	US	NV	S	(20.22; 2.90)	CS	DWS	c1a/c9a/w1d/c2a	CAAS/SSS/OES-S/WVS	0.61/0.26/0.44/0.41
Kim et al. (2020)	Korea and U.S.	DEC	655	US	NV	S	NA	CS	DWS	c1a/c9a/w1d/c2a	CAAS/SSS/OES-S/WVS	0.37/0.24/0.34/0.55
Kim et al. (2020)	Korea	DEC	420	GW	NV	E	(39.13; 9.26)	CS	DWS	c1a/c8a/w3b/w8a/c8b/c2a	CAAS/ED/MSQ/SWLS/WVS	0.46/−0.33/0.69/0.55/−0.39/ 0.15/
Kim and Kim (2022)	Korea	DEC	225	GW	NV	E	26–48 (36.66; 6.15)	CS	DWS	c8a/c2a	R-SSS/WVS	−0.56/0.48
Kozan et al. (2019)	Turkey	DEC	401	GW	NV	E	23–61 (39.30; 7.73)	CS	DWS	c1a/c9a/w3b/w8a/c2a	CAAS/PI-EA-SSS/JSI/SWLS/WVS	0.34/0.35/0.55/0.42/0.43
Ma et al. (2020)	China	DIC	854	US	NV	S	17−25 (20.21; 1.55)	CS	DWS	c1a/c3a/c8a/c2a	CAAS/CES/ECS/WVS	0.41/0.59/−0.14/0.59
Ma et al. (2021)	China	DIC	1231	FGCS	V	S	(20.19; 1.53)	CS	DWS	w1b/w3a/c1a/c8a/c2a	AES/ASS/CAAS/ECS/WVS	0.53/0.39/0.44/−0.11/0.61
Masdonati et al. (2019)	Switzerland	DEC	604	GW	NV	E	18–89 (41.80; 12.04)	CS	DWS	c9a/w3b/w8a/w5d/w5e/c7a/c2a	SS/JSS/SWLS/JIS	0.32/0.55/0.50/−0.47/−0.39/ −0.13/−0.53
McIlveen et al. (2021)	Australia	DEC	201	GW	NV	E	19–70 (42.39; 13.04)	CS	DWS	w1a/w1c/w3b/w4a/w1e	JSS/WIS/UWES	0.19/0.26/0.39/−0.33/0.36
Ribeiro et al. (2019)	Brazil	DIC	307	GW	NV	E	(42.83; 10.69)	CS	DWS	w3b/w10a/w4a	JSS/WAMI/WIS	0.55/0.34/−0.50
Rossier and Ouedraogo (2021)	Africa	DIC	501	GW	NV	E	7–78 (30.35; 15.24	CS	DWS	c9a/w3b/w10a/c7a/c2a	SC/JSS//WAMI/UH/WVS	0.15/0.48/0.27/0.16/0.39
Sheng and Zhou (2021)	China	DIC	361	GW	NV	E	(30.11;3.84)	CS	DWQ	c5a/c5b	TAW	0.38/0.25
Smith et al. (2020)	U.S.	DEC	240	SM	V	E	(29.02; 8.43)	LS	DWS	c8a/c2a	FSS/WVS	−0.65/0.50
Tokar and Kaut (2018)	U.S.	DEC	320	WD	V	E	18–63 (37.20, 9.46)	CS	DWS	c1a/c8a/c8b/c2a	CAAS/ECS/CIRDS/WVS	0.29/−0.65/−0.67/0.57
Vilhjálmsdóttir (2021)	Iceland	DEC	154	YLSW	V	E	18–29	CS	DWS	c1a	CAAS	0.23
Vollenhoven (2020)	Africa	DIC	139	DW	V	E	21- 69 (41.01; 11.21)	CS	DWS	c8a/c8b/c3b/c9b/c2a	ECS/LEMS/PPS/MSPSS/WVS	−0.20/0.08/0.24/0.28/0.49
Wang et al. (2019)	China	DIC	377	GW	NV	E	(33; 8.63)	CS	DWS	c9a/w3b/c3b/c9b/w4a/c2a	SSS/JSI/PPS/C-SSRS/TIS/WVS	0.32/0.60/0.22/0.35/−0.58/0.42
Wei et al. (2022)	China	DIC	254	USFIF	V	S	NA	CS	DWS	c1a/c9a/c8b/c3b/c2a	CAAS/SSS/LEMS/PPS/WVS	0.49/0.16/−0.06/0.45/0.57
Xu et al. (2022)	China	DIC	517	GW	NV	E	(34.0; 7.35)	CS	DWQ	c4a/w1f	IMS/ UWES	0.72/0.74

^a^Country; U.S., United States; P and B, Portugal and Brazil.

^b^Developed/developing countries; DEC, developed countries; DIC, developing countries.

^c^Population type; GW, general workers; US, university students; FGCS, first-generation college students; SM, sexual minorities; WD, workers with disabilities; USFIF, university students from impoverished families; LIW, low-income workers; DW, domestic workers; KW, knowledge workers; including teachers; researchers; and faculty members from universities; T, teachers; GWW, women workers from a wide spectrum of occupations; YLSW, young low-skilled workers.

^d^Vulnerable groups; V, vulnerable groups; NV, groups without specified vulnerabilities.

^e^Students/employees; S, students; E, employees.

^f^Study design; CS, cross-sectional studies; LS, longitudinal studies; Measure of DW; DWS, Decent Work Scale; DWQ, Decent Work Questionnaire.

^g^Outcome variables; C1a, career adaptability; C2a, work volition; C3a, career exploration; C3b, proactive personality; C4a, intrinsic motivation; C5a, learning factor of thriving at work; C5b, vitality factor of thriving at work; C6a, psychological capital; C7a, unemployment history; C8a, economic constraints; C8b, marginalization; C9a, economic resources; C9b, social support; w1a, absorption; w1b, academic engagement; w1c, dedication; w1d, occupational engagement; w1e, vigor; w1f, work engagement; w2a, burnout; w2b, cynicism; w2c, exhaustion; w2d, occupational fatigue; w2e, personal burnout; w2f, work fatigue; w3a, academic satisfaction; w3b, job satisfaction; w4a, turnover intention; w5a, basic needs satisfaction; w5b, needs satisfaction; w5c, psychological safety; w5d, qualitative job insecurity; w5e, quantitative job insecurity; w5f, survival needs satisfaction; w6a, community belonging; w6b, helping others; w6c, social contact need satisfaction; w6d, social contribution needs satisfaction; w6e, work family enrichment; w7a, self-determination needs satisfaction; w7b, psychological ownership; w8a, life satisfaction; w9a, health behaviors; w9b, health symptoms; w9c, physical health; w10a, meaningful work; 8. Measures for outcomes; WAMI, The Work and Meaning Inventory (Steger et al., 2012); WVS, Work Volition Scale (Duffy et al., 2012); LAMB, The Latent and Manifest Benefits of Employment (Muller et al., 2005); JSS, The Job Satisfaction Scale (Judge et al., 1998); SWLS, The Satisfaction With Life Scale (Diener et al., 1985); MNSS, Maslow's Safety/Security needs (Taormina and Gao, 2013); CAAS, the Career Adapt-Abilities Scale (Savickas and Porfeli, 2012); ECS, The Economic Constraints Scales (Duffy et al., 2019); LEMS, The Lifetime Experiences of Marginalization Scale (Duffy et al., 2019); FIPT, Family Income to Poverty Threshold (Pan et al., 2013); PM, The Perceived Marginalization (Issmer and Wagner, 2015); PCQ, The Psychological Capital Questionnaire (Luthans et al., 2015); WIS, The Withdrawal Intention Scale (Blau, 1985); OFER, The Occupational Fatigue Exhaustion Recovery Scale (Winwood et al., 2005); CBI, Copenhagen Burnout Inventory (Kristensen et al., 2005); SC, Social Class measured by two subjective items (Duffy et al., 2016); HHRDS, Heterosexist Harassment; Rejection; and Discrimination Scale (Szymanski, 2006); CFI, the Career Adaptability subscale from the Career Futures Inventory (Rottinghaus et al., 2005); HI-SC-SSS, Annual household income; social class; and subjective social status (Allan et al., 2014; Duffy et al., 2016; Autin et al., 2017); GEDS, The General Ethnic Discrimination Scale (Landrine et al., 2006); WNSS, Work Need Satisfaction Scale (Autin et al., 2019); FPAQ, The Food and Physical Activity Questionnaire (Murray et al., 2017); PHQ, The Patient Health Questionnaire—Somatic Symptom Scale (Kroenke et al., 2002); WFI, Work Fatigue Inventory (Frone and Tidwell, 2015); UWES, The Utrecht Work Engagement Scale (Schaufeli et al., 2006); MBI-GS, MBI–General Survey (Schaufeli et al., 1996); BNS, Basic Needs Satisfaction (Deci et al., 2001); PSS, Psychological Safety Scale (Edmondson, 1999); JSI, Job Satisfaction item; WFE, Work-Family Enrichment (Carlson et al., 2006); SSS, The MacArthur Scale of Subjective Social Status (Adler et al., 2000); OES-S, Occupational Engagement Scale for Students (Cox, 2008); ED, Economic Difficulties (Kang, 2016); MSQ, The Minnesota Satisfaction Questionnaire (Park, 2005); PI-EA-SSS, Personal income; educational attainment; and one-item MacArthur Scale of Subjective Social Status (Adler and Stewart, 2007); CES, Career Exploration Scale; AES, The Academic Engagement Scale (Reeve and Tseng, 2011); ASS, Academic Satisfaction Scale (Lent et al., 2007); SS, Social Status; JIS, Job Insecurity Scale; TAW, Thriving at Work Scale (Porath et al., 2012); FSS, Financial Strain Scale (Creed and Macintyre, 2001); MSPSS, Perceived Social Support (Zimet et al., 1988); C-SSRS, Chinese Social Support Rating Scale (Xiao, 1994), TIS, The Turnover Intention Scale (Mobley et al., 1978); IMS, intrinsic motivation scale (Dewett, 2007; Grant, 2008). R-SSS, Reversed calculation of the MacArthur Scale of Subjective Social Status; PPS, Proactive Personality Scale (Bateman and Crant, 1993).

Regarding methodology, all included studies were conceptually based on a psychological perspective emphasizing measuring decent work from the perspective of the individual participants, among which 39 studies used the Decent Work Scale developed by Duffy et al. (2017), and seven studies used the Decent Work Questionnaire developed by Ferraro et al. (2018) to measure decent work. Most of the included studies were cross-sectional studies (*n* = 41). The instruments used for measuring variables of wellbeing and career capabilities varied across different studies; yet some scales were identified to be more frequently used among the selected studies: the Work and Meaning Inventory (WAMI) (Steger et al., 2012) was frequently used for measuring meaningful work (*n* = 10), the Work Volition Scale (WVS) (Duffy et al., 2012) for work volition (*n* = 21), the Job Satisfaction Scale (JSS) (Judge et al., 1998) for job satisfaction (*n* = 10), the Satisfaction With Life Scale (SWLS) (Diener et al., 1985) for life satisfaction (*n* = 7), the Career Adapt-Abilities Scale (CAAS, Savickas and Porfeli, 2012) or the Career Futures Inventory (CFI) (Rottinghaus et al., 2005) for career adaptabilities (*n* = 13 and *n* = 4 respectively), the Economic Constraints Scales (ECS) (Duffy et al., 2019) for economic constraints (*n* = 8), the Lifetime Experiences of Marginalization Scale (LEMS) (Duffy et al., 2019) for marginalization (*n* = 6), the Withdrawal Intention Scale (WIS) (Blau, 1985) for turnover intention (*n* = 7), the Copenhagen Burnout Inventory (CBI) (Kristensen et al., 2005) for burnout (*n* = 4), the Utrecht Work Engagement Scale (UWES) (Schaufeli et al., 2006) for work engagement *(n* = 7), and the MacArthur Scale of Subjective Social Status (SSS) (Adler et al., 2000) for economic resources (*n* = 7), etc.

### Subgroups of included studies

The included studies were categorized into different subgroups in accordance with six dimensions, namely developmental status of countries as developed or developing, population type, social status of participants as employee or student, participants from vulnerable or general groups, study design, and study variables of wellbeing and/or career capabilities. All classifications of the included studies are presented in Table 1 except the last one concerning the study variables of wellbeing/career capabilities, which is presented in Table 2. First of all, according to the Human Development Index (HDI) used by the United Nations (World Bank, 2022), we classified the data collection sites of the included studies to be developing or developed countries. It turned out that 14 included studies were conducted in developing countries (i.e., three studies in Africa, eight in China, one in India, and two in Brazil), 30 in developed countries (i.e., one in Australia, one in Iceland, three in Italy, two in Portugal, one in Switzerland, three in Turkey, three in South Korea, 15 in the United States, and one in the both United States and South Korea), and two studies in both developed and developing countries (i.e., Portugal and Brazil).

**Table 2:** Recategorization of study variables and indicators of wellbeing and career capabilities in the included studies.

**Recategorization of study variables**	**Study variables**	**Indicator(s) used in the selected studies (codes of the indicators)**
**Wellbeing**		
Positive wellbeing	Engagement	Absorption (w1a); academic engagement (w1b); dedication (w1c); occupational engagement (w1d); vigor (w1e); work engagement (w1f)
	Work satisfaction	Academic satisfaction (w3a); job satisfaction (w3b)
	Meaningful work	Meaningful work (w10a)
	Life satisfaction	Life satisfaction (w8a)
	Positive states of health	Health behaviors (w9a); physical health (w9c)
Negative wellbeing	Burnout/fatigue	Burnout (w2a); cynicism (w2b); exhaustion (w2c); occupational fatigue (w2d); personal burnout (w2e); work fatigue (w2f)
	Turnover intention	Turnover intention (w4a)
	Negative states of health	Health symptoms(w9b)
Needs satisfaction	Survival needs satisfaction	Basic needs satisfaction (w5a); maslow's security/safety need satisfaction (w5b); psychological safety (w5c); qualitative job insecurity (w5d); quantitative job insecurity (w5e); survival needs satisfaction (w5f)
	Social connection needs satisfaction	Community belonging (w6a); helping others (w6b); social contact need satisfaction (w6c); social contribution needs satisfaction (w6d); work family enrichment (w6e)
	Self-determination needs satisfaction	Self-determination needs satisfaction (W7a)
**Career capabilities**		
Psychosocial resources	Career adaptability	Career adaptability (c1a)
	Work volition	Work volition (c2a)
	Proactivity	Career exploration (c3a); proactive personality (c3b)
	Motivation	Intrinsic motivation (c4a)
	Thriving at work	Learning factor of thriving at work (c5a); vitality factor of thriving at work (c5b)
	Psychological capital	Psychological capital (c6a)
	Environmental resources	Economic resources (c9a); social support (c9b)
Psychosocial constraints	Unemployment history	Unemployment history (c7a)
	Environmental constraints	Economic constraints (C8a); marginalization (C8b)

Second, according to the occupations of the participants, we categorized all participants into four types encompassing knowledge workers (i.e., faculty members from universities, researchers, teachers, and high-skilled adults, *n* = 6), general workers from a wide spectrum of occupations (*n* = 28), relatively underprivileged groups (i.e. low-income workers, sexual minorities, domestic workers, workers with disabilities, university students from impoverished families, young low-skilled workers, and first-generation college students, *n* = 9), and university students without specific vulnerabilities (*n* = 3). Third, the included studies were also categorized into two subgroups of students (*n* = 5) versus employees (*n* = 41) based on their social status. Fourth, the included studies were also classified as studies focused on vulnerable versus non-vulnerable groups, the former of which involved participants with specific vulnerabilities (i.e., low-income workers, domestic workers, people with disabilities, sexual minorities, young low-skilled workers, university students from impoverished families, and first-generation college students, *n* = 9), and the latter involved participants without specific vulnerabilities (*n* = 35). Moreover, with respect to study design, five studies were longitudinal whereas 41 studies were cross-sectional.

Finally, we classified the study variables of wellbeing and career capabilities in these included studies and identified ten study variables to be under the umbrella of wellbeing (i.e., engagement, burnout, job satisfaction, turnover intention, survival needs satisfaction, social connection needs satisfaction, self-determination needs satisfaction, and meaningful work), and nine study variables of career capabilities (i.e., career adaptability, work volition, proactivity, motivation, thriving at work, psychological capital, unemployment history, environmental constraints, and environmental resources). Each identified variable may be measured by different indicators. All the study variables and indicators were recategorized into different aspects of wellbeing (i.e., positive wellbeing, negative wellbeing, and needs satisfaction), and career capabilities (i.e., psychosocial resources and psychosocial constraints).

### Association of decent work with wellbeing

**Figure 2:**
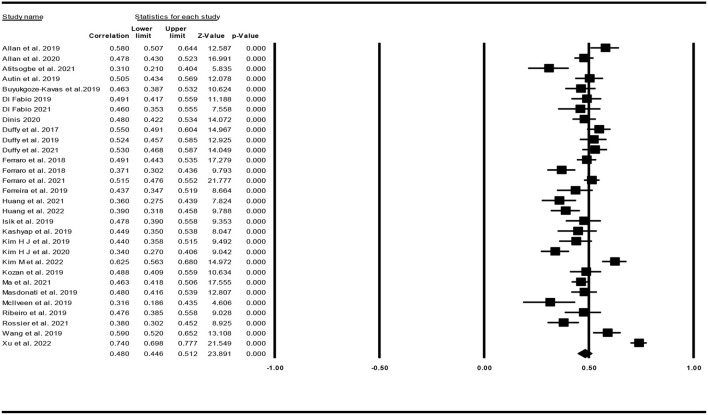
Effect sizes of each study regarding the association between decent work and wellbeing.

The effect sizes of each study regarding the association between decent work and wellbeing are presented in Figure 2. The mean correlation coefficient between decent work and wellbeing was .48 with a 95% confidence interval of.45 to.51. The results of heterogeneity tests supported a significant heterogeneity among the studies (Q = 219.96, df = 29, *p* < 0.001, I^2^ = 86.82). We calculated the prediction interval using the mean effect size (.48), upper limit of confidence interval (0.512), Tau^2^ (.0122) and number of studies (30) and it turned out that the true correlation coefficient in 95% of all comparable populations fell in the interval 0.28 to 0.64.

### Association of decent work with career capabilities

**Figure 3:**
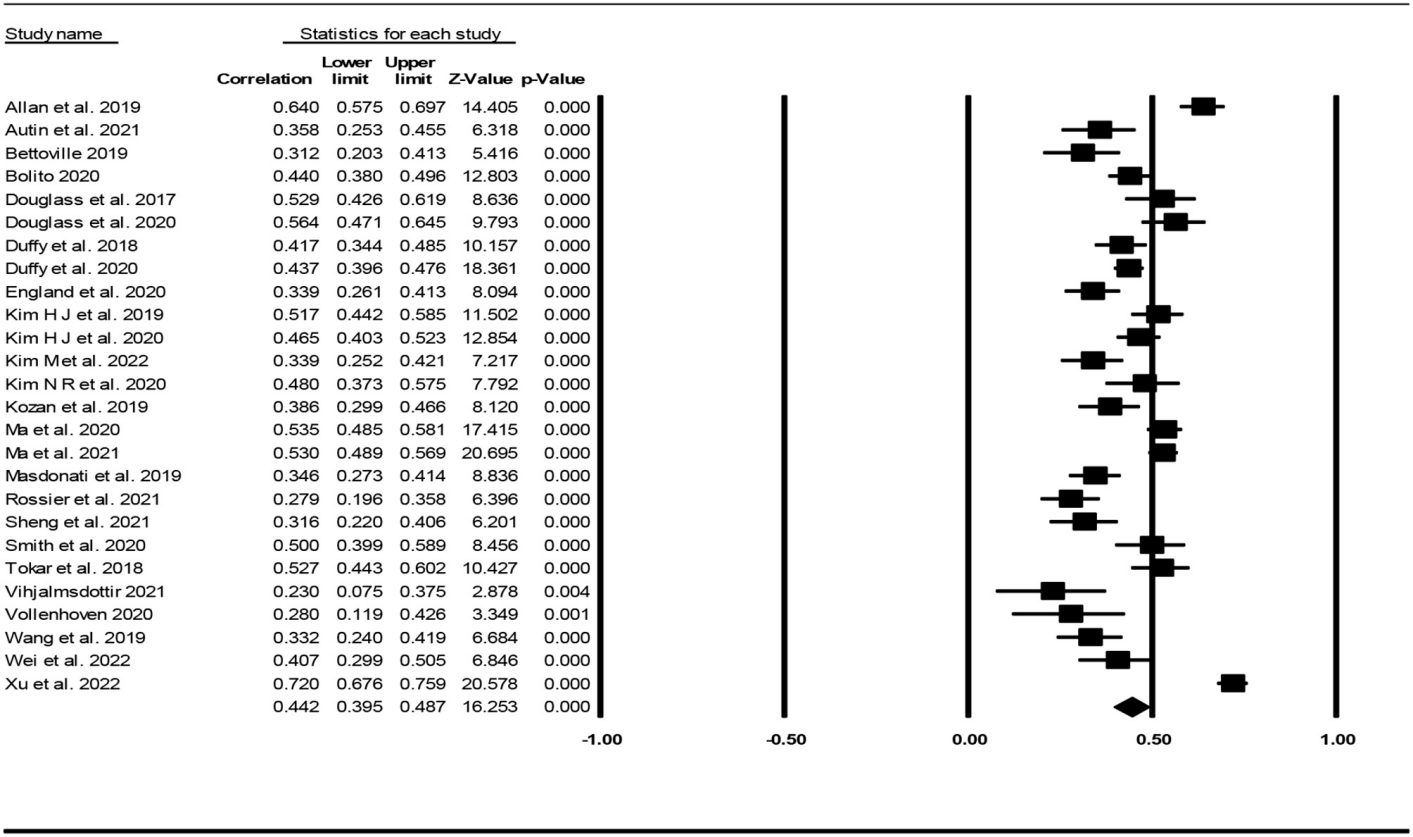
Effect sizes of each study regarding the association between decent work and career capabilties.

The effect sizes of each study regarding the association between decent work and capabilities are presented in Figure 3. The mean correlation coefficient between decent work and career capabilities was .44 with a 95% confidence interval of.40 to.49. There was also a significant heterogenity among the studies (Q = 250.21, df = 25, *p*<*0.001*, I^2^ = 90.01). Using the mean effect size (.44), upper limit of confidence interval (0.49), Tau^2^ (0.0194) and number of studies (26), the prediction interval representing the true correlation coefficient in 95% of all comparable populations was calculated to fall in the interval 0.18 to 0.65.

### Subgroup comparisons

Table 3 presents the results of group comparisons with respect to the association of decent work with wellbeing and career capabilities. As all *p-*values for between-group heterogeneity are larger than .05, it turned out that no significant differences were identified across all subgroups which are categorized by developed/developing countries, population type of knowledge workers, relatively underprivileged workers, general workers or university students, participants with or without specific vulnerabilities, social status of participants as employee or student, the recategorized outcome variables, and the study design of cross-sectional versus longitudinal studies. Specifically speaking, decent work demonstrated medium levels of association with different variables under the umbrella of wellbeing and career capabilities across different contexts characterized by the developmental status of the countries, population type, vulnerabilities of participants, and social status of the participants.

**Table 3:** Subgroup analyses for the associations of decent work with wellbeing and career capabilities.

	**Wellbeing**							**Career capabilities**								
**Subgroups**	**K**	**ES**	**LL**	**UL**	**p** ^a^	**Q**_w_	**Q**_b_	**p** ^b^	**K**	**ES**	**LL**	**UL**	**p** ^a^	**Q**_w_	**Q**_b_	**p** ^b^
**Developed/developing countries**							1.21	0.54							0.16	0.92
Developed countries	18	0.49	0.47	0.50	0.000	61.24			16	0.43	0.41	0.45	0.000	89.09		
Developing countries	10	0.48	0.46	0.50	0.000	142.06			8	0.49	0.46	0.51	0.000	148.92		
Both	2	0.42	0.37	0.46	0.000	9.79			2	0.45	0.41	0.49	0.000	0.34		
**Population types**							6.38	0.09							5.71	0.06
Knowledge workers^a^	6	0.46	0.44	0.49	0.000	27.62			N.A.	N.A.	N.A.	N.A.	N.A.	N.A.		
Relatively underprivileged groups^b^	3	0.50	0.47	0.53	0.000	8.00			8	0.51	0.48	0.54	0.000	46.00		
General workers^c^	19	0.49	0.48	0.51	0.000	162.23			15	0.42	0.40	0.44	0.000	161.09		
University students	2	0.38	0.33	0.43	0.000	3.48			3	0.51	0.47	0.54	0.000	3.31		
**Participants with/without specific vulnerabilities**							1.28	0.26							0.79	0.38
Non-vulnerable group	27	0.48	0.46	0.49	0.000	213.74			18	0.44	0.42	0.45	0.000	184.84		
Vulnerable group^d^	3	0.50	0.47	0.53	0.000	8.00			8	0.46	0.43	0.49	0.000	46.00		
**Social status of participants**							2.63	0.11							0.14	0.71
Employee	27	0.49	0.48	0.50	0.000	198.42			21	0.43	0.41	0.45	0.000	217.35		
Student	3	0.43	0.39	0.46	0.000	9.35			5	0.51	0.48	0.53	0.000	8.56		
**Outcome variables**							0.64	0.73							2.74	0.10
Positive wellbeing	10	0.49	0.47	0.51	0.000	159.66			N.A.	N.A.	N.A.	N.A.	N.A.	N.A.		
Negative wellbeing	13	0.50	0.48	0.51	0.000	34.07			N.A.	N.A.	N.A.	N.A.	N.A.	N.A.		
Needs satisfaction	7	0.46	0.43	0.48	0.000	40.96			N.A.	N.A.	N.A.	N.A.	N.A.	N.A.		
Psychosocial resources	N.A.	N.A.	N.A.	N.A.	N.A.	N.A.			9	0.45	0.33	0.55	0.000	149.99		
Psychosocial constraints	N.A.	N.A.	N.A.	N.A.	N.A.	N.A.			17	0.33	0.24	0.41	0.000	307.00		
**Study design**							0.10	0.75							0.10	0.76
Cross-sectional study	27	0.49	0.48	0.50	0.000	207.61			24	0.46	0.44	0.47	0.000	248.65		
Longitudinal study	3	0.46	0.42	0.49	0.000	31.86			2	0.45	0.41	0.48	0.000	1.35		

^a^Knowledge workers for decent work and wellbeing include faculty members from universities, researchers, teachers, and high-skilled adults.

^b^For decent work and wellbeing, relatively underprivileged groups (RUG) include first-generation college students, low-income workers, and sexual minorities, whereas for decent work and career capabilities, RUG include domestic workers, first-generation college students, workers with disabilities, sexual minorities, university students from impoverished families, and young low-skilled workers.

^c^General workers refer to workers from a wide spectrum of occupational backgrounds ranging from high-skilled to low-skilled jobs.

^d^Vulnerable groups include all those relatively underprivileged groups (RUG). K, number of studies; ES, effect size; LL, lower limit; UL, upper limit; p, p value for effect size; pb, *p*-value for between group heterogeneity; Q_w_, Q(within); Q_b_, Q(between); N.A, refers to non-applicable.

### Publication bias

**Figure 4:**
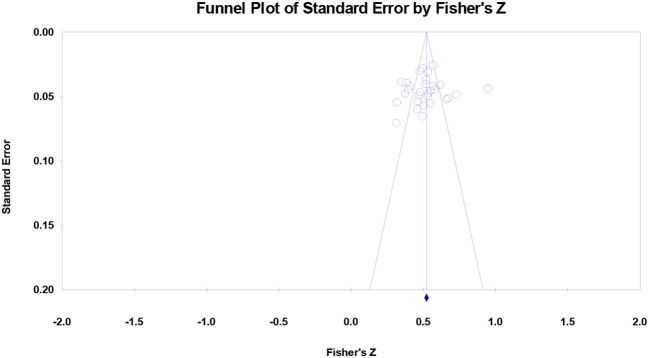
Funnel plot of effect sizes for the association of decent work with wellbeing. Note that the mean of combined outcome variables was used.

**Figure 5:**
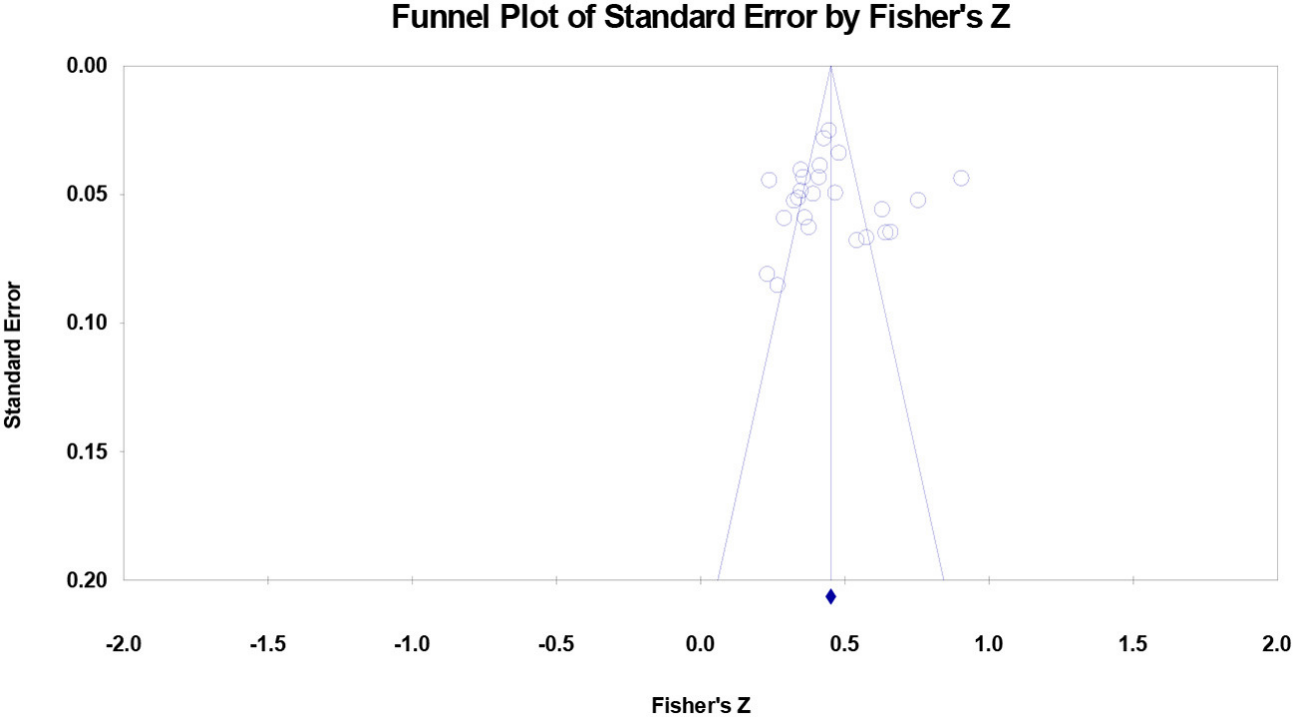
Funnel plot of effect sizes for the association of decent work with career capabilities. Note that the mean of combined outcome variables was used.

All of the studies distributed symmetrically around the combined effect sizes for the association between decent work and wellbeing (Figure 4) and for the association between decent work and career capabilities (Figure 5) and none of the studies fell at the bottom of the graphs. The symmetrical shapes of the funnel plots suggest no publication bias.

## Discussions

This meta-analysis examined empirical findings about the mean associations of decent work with wellbeing and career capabilities and tested the heterogeneity of the associations across different contexts. The results indicate medium levels of mean associations of decent work with both wellbeing and career capabilities, and no significant differences in the associations in subgroups in accordance with the developmental status of countries and the variety of population groups. These findings can help draw theoretical, political, social, and research implications as follows.

First, this meta-analysis enriches our understanding about the notion of decent work, its applications in different contexts, and its diverse correlates. The included studies supported decent work as an ideal state of current work conditions as well as a work-related aspiration at the personal level, and a policy objective at the macro level, and decent work can exist among employees as well as university students, the latter of which may develop perceptions of decent work, which could influence their present academic engagement, academic satisfaction, work volition, and career adaptability (Ma et al., 2020, 2021). The included studies also revealed that those people with specific vulnerabilities such as those who are sexual minorities, low-income workers, or students from impoverished families may develop decent work perceptions regardless of the constraints facing their work and life (Allan et al., 2019; Duffy et al., 2019; Wei et al., 2022). In addition to concerning the association of decent work with people's wellbeing, the included studies also revealed the associations of decent work with a wide spectrum of study variables under the umbrella of career capabilities as informed by the capability approach, and these findings are deemed conducive to developing new conceptual frameworks or enriching existing conceptual frameworks for explicating employees' or students' sustainable career development. For example, a conceptual framework is needed for explicitly linking up decent work and career capabilities manifested in terms of both psychosocial resources and psychosocial constraints, which are conceptually deemed important for developing a sustainable career. Such a conceptual framework may ride on the psychology of working theory (Blustein et al., 2016, 2019, 2023), and help expand the capacity of the latter with a psychosocial perspective in relation to explicating in what way the promotion of decent work can lead to sustainable positive outcomes in organizational contexts in a long-term manner through strengthening people's career capabilities (Su et al., 2022). Such a conceptual framework may inform relevant research and practice for substantiating the benefits of promoting decent work in organizational contexts. Theoretically speaking, such a conceptual framework can also help inform the development of the agency-structure theory (Giddens, 1979, 1984) as well as the sustainable career conceptualization (Van der Heijden and De Vos, 2015). According to the agency-structure theory, people's agentic practice is shaped by structures and their practices also further constitute structures. Consistent with the logic of agency-structure theory, the suggested framework denotes that decent work as social structures and career capabilities as exercise of human agency (i.e., enhancing psychosocial resources and reducing psychosocial constrains) can presuppose each other. Notably, this framework may add values to the agency-structure theory by situating the latter in a context of pursuing a sustainable career, which provides broader temporal and spatial dimensions for enriching the relationship between agency and structures. Moreover, such a conceptual framework can also help scale down “individualism” in the conceptualization of sustainable career (Bal et al., 2020), and opt for developing an enabling and empowering environment for enhancing people's exercise of agency in their career development.

Second, this meta-analysis reported overall medium levels of mean associations of decent work with both wellbeing and career capabilities. Conceptually decent work has been argued to play an important role in promoting wellbeing and career development (Blustein et al., 2016; Duffy et al., 2016). These findings are consistent with a prior literature review suggesting that promoting decent work will lead to positive impacts in organizational contexts (Pereira, 2019). Moreover, the findings suggest conclusive estimates regarding the magnitudes of the associations between decent work with wellbeing and career capabilities, which resulted in biases caused by the sparse findings of previous studies conducted in various contexts.

Another important finding of this study relates to the subgroup comparisons with respect to investigating the influence of different study contexts on the associations of decent work with wellbeing and career capabilities. Although the decent work notion has emerged for more than two decades, the practice of promoting decent work varies across different contexts. It is assumed that some factors such as the developmental status of countries may affect the effects of promoting decent work. For example, in developing countries where working conditions are usually jeopardized by the deprivation of social and economic resources, how to position the goal of promoting decent work remains a controversial topic (Singh, 2017; Dhakal and Burgess, 2021). In China, the goal of achieving decent work is still at an early stage and the majority of the public are not able to associate the decent work notion with their present or future work (Cooke et al., 2019). Scientific evidence is required to justify the importance of promoting decent work in developing countries and across diverse underprivileged population groups. The results of this meta-analysis demonstrate that no significant differences with respect to the moderate association of decent work with wellbeing and career capabilities were identified across different contexts characterized by the developmental status of the countries, variety of population type, vulnerabilities of participants, and social status of the participants. These findings justify the actions taken to speed up the advocacy of decent work across the world and among different population groups.

To our surprise, the results show that heterogeneities were identified in the mean effect sizes in relation to the associations of decent work with both wellbeing and career capabilities; yet their corresponding subgroup comparisons were all insignificant. There remain chances that some factors that can explicate the overall heterogeneities were not identified in this meta-analysis. The categorizations of subgroups in this meta-analysis are bounded by the availability of relevant correlations in the included studies. We classified population type into four subgroups, namely knowledge workers, relatively underprivileged groups, general workers and university students. It is obvious that the subgroup of general workers, which encompasses employees from a wide spectrum of occupational backgrounds ranging from high-skilled to low-skilled jobs have overlaps with other subgroups. This is related to the fact that many included studies opted to report the associations of decent work with wellbeing and career capabilities among general workers rather than report the correlations in subgroups. Moreover, the social status of the participants in the included studies was employee or student, and no included studies focused on people who are not in education, employment or training. In addition, only one included study focused on workers with disabilities, which may increase the challenges to identify the heterogeneity in subgroup comparisons. To identify the factors associated with the heterogeneity in the overall mean effect sizes, it is suggested to conduct studies to test the associations of decent work with wellbeing and/or career capabilities among diverse subgroups.

Last but not least, this meta-analysis may contribute to more discussions regarding the operationalization of decent work. Decent work in the included studies was mainly measured by using the self-reported instruments of Decent Work Scale or the Decent Work Questionnaires highlighting the subjective experiences or perceptions of decent work in the eyes of the beholders. The findings of this meta-analysis study support the importance of discerning and promoting subjective experiences of decent work experiences or perceptions of decent work with respect to enhancing wellbeing and career capabilities. When compared with Webster's tool, which uses dichotomous questions, Duffy's and Ferraro's instruments have two distinctive features as revealed by the included studies. First, the Likert scale questions used by these two instruments are favorable for conducting advanced statistical analyses. Second, the psychological perspective underlying these two instruments is conducive to linking with other psychological concepts or conceptual frameworks. The decent work notion developed by Duffy, which was informed by the psychology of working theory, has been given a broad psychosocial perspective and has sparked a keen academic interest in studying the influence of decent work in organizational contexts (Duffy et al., 2019; Autin et al., 2021). Ferraro's decent work notion which highlights individuals' perceptions of work by referring to the ILO's 11 substantive elements, has also been studied in conjunction with a wide variety of psychological phenomena, such as personal burnout, work engagement, thriving at work, and motivation, etc. (Ferraro et al., 2021; Sheng and Zhou, 2021; Xu et al., 2022).

### Limitations

The findings of this meta-analysis are confined to research designs and targeted population groups of the included studies. First, most of the included studies are cross-sectional studies. In the long run, it is suggested to conduct more longitudinal studies with respect to decent work and its association with wellbeing and career capabilities for drawing conclusions about causal relationships. Second, insignificant results of subgroup comparisons may emerge because of the unavailability of relevant correlations in the included studies. Thus, it is suggested to enrich the diversity of the targeted participants by conducting studies to test the associations of decent work with wellbeing and/or career capabilities among more subgroups, including those working in high-skilled versus low-skilled jobs, participants who are not in education, employment or training, and workers who are suffering from different types of vulnerabilities. Finally, the findings of this study may be confined by the participants' subjective perceptions, as all included studies measured decent work by using self-reported measures. As decent work can be taken as an ideal state of current work conditions as well as a work-related aspiration to be realized at the personal level, and a policy objective to be achieved at the macro level, it is important to investigate the association of decent work with wellbeing and career capabilities by using both objective and subjective indicators for measuring and promoting decent work across different countries and various population groups.

## Data availability statement

The raw data supporting the conclusions of this article will be made available by the authors, without undue reservation.

## Author contributions

XS was responsible for designing the study, searching literature, conducting data analysis, drafting, and revising the article. KLC was responsible for supervising the study and reviewing the draft of the article. All authors contributed to the article and approved the submitted version.
